# Rectal prolapse in children: Laparoscopic suture rectopexy is a suitable alternative

**DOI:** 10.4103/0971-9261.70634

**Published:** 2010

**Authors:** Bipin Puri

**Affiliations:** Consultant, Surgery and Pediatric Surgery, Army Hospital (R and R), Delhi, India

**Keywords:** Rectal prolapse, laparoscopy, rectopexy

## Abstract

**Aim:**

To study the clinical outcome of laparoscopic suture rectopexy (LSRP) in children with persistent rectal prolapse (PRP).

**Materials and Methods:**

Nineteen cases of PRP were managed with LSRP from February 2005 to August 2009.

**Results:**

All were followed up for an average duration of 6 months. Only one child had recurrence and was managed with sclerotherapy.

**Conclusions:**

LSRP is safe, feasible in children and gives satisfactory results.

## INTRODUCTION

Rectal prolapse is a relatively common, usually selflimiting illness in children. Peak incidence is between 1 and 3 years.[1,2] It can be partial (protrusion of only mucosa from anal verge) or complete (full thickness of rectum is involved). The intervention is required for the persistent rectal prolapse (PRP). Laparoscopic suture rectopexy (LSRP) is in vogue for adults; however, only scanty experience is available with this technique in children. We present our experience with LSRP for PRP at the pediatric surgical center at Army Hospital (R and R), Delhi.

## MATERIALS AND METHODS

This is a retrospective analysis of 19 children managed with LSRP for PRP from February 2005 to August 2009. The conservative management of nutritional support, laxatives, dietary manipulation and sclerotherapy for managing the prolapse had failed in all and were referred for surgical intervention. Four of the 19 patients were managed with sclerotherapy using Polidocanol injected submucosally in three to four sittings at peripheral hospitals before being referred to this center for surgical intervention. Cases with rectal prolapse who did not respond to conservative management over 1 year were defined as PRP and were subjected to LSRP. The decision to operate was based on the age of patient, duration of conservative management (>12 months) and frequency of recurrent prolapse [two or more episodes requiring manual reduction under sedation/ general anesthesia (GA)], along with symptoms of pain, rectal bleeding and recurrent prolapse. A record of age, sex, weight, initial presentation, duration of symptoms, precipitating events and co-morbidities was maintained. Preoperative evaluation included physical examination, routine laboratory investigations, barium enema and proctoscopy in all patients. The parents were informed about the procedure and all consented for the same.

All were given enemas on the morning of the surgery. Prophylactic antibiotics were given at the time of induction. All were operated under GA with endotracheal intubation. Veress needle capnoperitoneum was established through umbilical incision to create intrabdominal pressure of 10 mmHg. This was followed by introduction of 5-mm, 30° scope at umbilicus and two 5-mm working ports in midclavicular line over the line joining mid-inguinal point and both costal margins. The position of the working ports varied with the height of the child, ensuring triangulation and a reasonable play of the port instruments. Trendelenburg position helped in moving away the small bowel from the operative field.

The rectosigmoid was grasped and mobilized after dividing peritoneum circumferentially. Both the ureters were identified and safeguarded. Rectum was mobilized from the sacral promontory up to the lateral ligaments, and until the surface of the sacrum was clearly felt with an instrument. Rectum was then pulled up and fixed with the presacral fascia on either side with three to five seromuscular sutures of braided silk size 3/0 using intracorporeal knotting. Rectum was reperitonealized after ensuring a satisfactory fixation. Patients were kept nil orally till the return of bowel sounds. Postoperatively, stool softeners were routinely prescribed for at least 2 weeks.

## RESULTS

Of the 19 children, 13 (68.82%) were males and 6 (31.18%) were females. Male to female ratio was 2:1. The mean age of presentation was 5 years (range 3–8 years). The presenting complaints were mass descending per rectum along with bleeding per rectum lasting from 1 to 2 years. All had rectal prolapse of 3–5 cm in length. Two children were malnourished and one had cystic fibrosis. The two children who were malnourished were both males and weighed 12.4 kg and 15.2 kg at ages 5 years and 7 years, respectively, and were noted to be below the 5^th^ centile as per NCHS weight for age charts. The child with cystic fibrosis was a female aged 6 years and weighed 13.8 kg which was below the 5th centile as per NCHS weight for age charts. The remaining 16 out of 19 children were normal in weight and fell between the 20th and 50th centile by NCHS standards. The patient profile is depicted in Table 1.

**Table 1:** Patient profile

No. of patients	19
Males [*n* (%)]	13 (68.82%)
Females [*n* (%)]	6 (31.18%)
Length of prolapse	3–5 cm
Presenting complaints	Mass descending per rectum: 19
	Bleeding per rectum: 15
Co-morbid conditions	Malnourished: 2
	Cystic fibrosis: 1
Precipitating factors	Bout of diarrhea: 2

The mean duration of surgery was 75 minutes (range 60–120 minutes). No intraoperative complications were repor ted. Redundancy of rectosigmoid was noticed in all patients except the two with malnutrition. Pelvic floor laxity was not found in any. No intraoperative problems were encountered and no case required conversion. Mean postoperative hospitalization was 6 days (range 4–10 days). All were followed up for an average of 6 months (range 4–12), with recurrence reported in one case with cystic fibrosis. This was partial prolapse of rectal mucosa which was dealt with three sittings of sclerotherapy using inj. Polidocanol. One child complained of postoperative constipation which improved with dietary manipulation and stool softeners.

## DISCUSSION

The exact etiology of rectal prolapse in children is unknown. It is thought to be related to several anatomic considerations such as the vertical configuration of the sacrum, greater mobility of the sigmoid colon, a loosely attached rectal mucosa to the underlying muscularis, and the absence of Houston’s valves in approximately 75% of infants younger than 1 year of age.[1] Patients with rectal prolapse have lower basal and squeeze pressures with anorectal manometry than normal control subjects.[3,4] Rectal prolapse usually presents as a self-limiting disorder in children younger than 4 years of age.[5,6] In the pediatric population, the condition is usually diagnosed by the age of 3 years, with an equal sex distribution.[7] Male preponderance has been noted by Shalaby *et al*.[8] and our study reaffirmed a male preponderance with 70% of patients being males.

Mostly conservative treatment is successful;[6] however, the prolapse may persist indefinitely in some children, requiring surgical intervention. The percentage of children requiring surgical intervention, eventually, after failure of conservative management varies from 14 to 20%.[9] Surgery is indicated in rare cases with intractable rectal prolapse and may be considered in patients who are not spontaneously cured in 12–18 months of follow-up.[9] The mean period of conservative management in our study could not actually be ascertained as this study was conducted at a tertiary care hospital, whereas the patients were managed at peripheral hospitals for a variable period of time. However, a trial of at least 12 months of conservative management was given at the peripheral hospitals before the patients were referred to our center. Two patients had to be operated earlier than the mandatory period of 12 months as they reached the other end point of recurrent prolapse requiring manual rectal repositioning under GA.

Literature is replete with various procedures for this condition which is a testimony to the lack of consensus over an ideal procedure. Broadly, the operative procedures can be classified as abdominal[10] or perineal.[11–15] Less invasive procedures include injection sclerotherapy[6,16,17] and encircling of the anus,[18] with reported success rate of nearly 90% in different series.

Abdominal rectopexy is advocated for the recurrent or PRP in children. In adults, in a recent meta-analysis comparing outcomes using the laparoscopic technique with an open procedure, no differences in operative morbidity and recurrence rates were found.[19]

As experience is being gained in the pediatric cases with the laparoscopic approach, it has been shown to have good results.[9,20,21] Laparoscopic surgery has the advantages of less pain, shorter hospital stay and early recovery, as compared with laparotomy. Apart from these advantages, the results are similar to those with the open procedures irrespective of the method used (suture, resection or posterior mesh). Therefore, where expertise is available, this approach may be preferred.[7]

Koivusalo *et al*. reported a median operation time of 80 minutes (range 62–90 minutes) for LSRP and a median hospital time of 6 days (range 3–8 days).[9] Shalaby *et al*., in their study, reported the mean duration of surgery as 40 minutes (range 30–55 minutes). The mean hospitalization time was 3 days.[8] Experience with LSRP in this study further reinforces these findings. The mean duration of surgery was 75 minutes (range 60–120 minutes). No intraoperative complications were reported. Mean postoperative hospitalization was 6 days (range 4–10 days).

The recurrence rates reported for PRP are as much as 6.9% at 5 years and 10.8% at 10 years.[8] Recurrent cases can be treated by laparoscopic resection rectopexy with or without mesh.[17,21] However, Rintala and Pakarinen prefer laparoscopic suspension of the rectum to anterior sacrum without mesh and they claimed that this approach is successful in several patients.[9] In this study, after a mean follow-up of 6 months, we had one recurrence in the form of partial rectal prolapse in a child with cystic fibrosis which was managed by sclerotherapy

Koivusalo[9] reported two patients with postoperative constipation. They added that constipation is the only postoperative problem and is frequently worsened. Shalaby *et al*. reported only one case of postoperative constipation out of 52 cases operated with laparoscopic mesh rectopexy.[8] In our study too, we had just one case of postoperative constipation which could be managed conservatively. This stands in stark contrast to high rate (35%) of postoperative constipation reported earlier by Kariv *et al*. All our 19 children were bowel continent at the time of presentation and none of them had any continence issues in the postoperative setting.

Although this is a single center experience without a control group, the results are satisfying. Whereas larger randomized control studies are required to secure conclusive evidence for the superiority of LSRP over the conventional open procedure, paucity of PRP cases in a single center remains the limiting factor. We conclude that LSRP is an effective and safe alternative to the open procedures with similar success rates and no additional complications.
